# Unmasking vertebral artery stump syndrome in recurrent posterior strokes treated with endovascular therapy

**DOI:** 10.1055/s-0045-1806833

**Published:** 2025-06-01

**Authors:** Leonardo Furtado Freitas, Kevin J. Abrams, Márcio Luís Duarte, Guilherme C. Dabus

**Affiliations:** 1 Baptist Health of South Florida, Radiology Associates of South Florida, Miami FL, United States.; 2 Florida International University, Herbert Wertheim College of Medicine, Miami FL, United States.; 3 Baptist Health South Florida, Department of Radiology, Division of Clinical Neuroradiology, Miami FL, United States.; 4 Universidade de Ribeirão Preto, Departamento de Radiologia, Guarujá SP, Brazil.; 5 Diagnósticos da América S.A., São Paulo SP, Brazil.; 6 Baptist Health South Florida, Department of Radiology, Division of Interventional Neuroradiology, Miami FL, United States.

**Figure 1:**
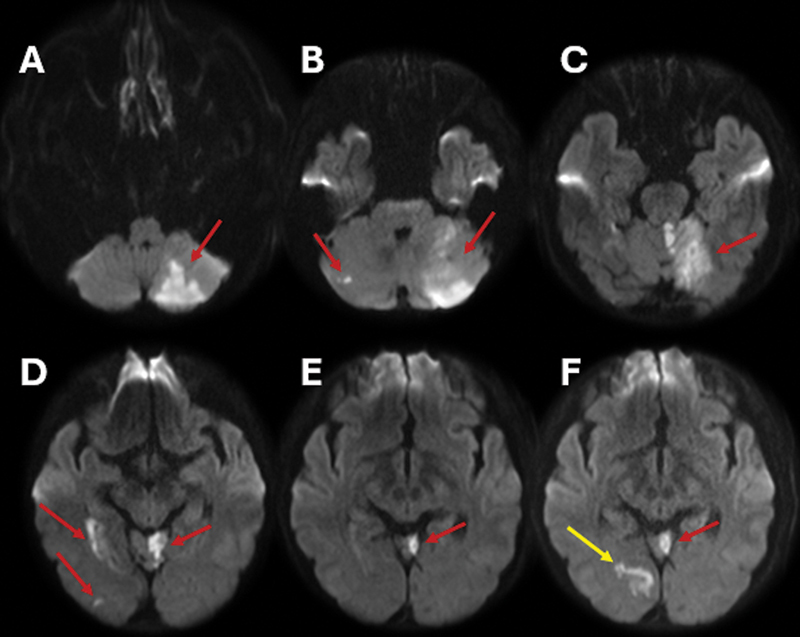
Magnetic resonance imaging (MRI) scan of the brain, axial diffusion sequence. ( **A–E** ) Multiple acute/subacute infarcts in the posterior circulation (red arrows), involving the cerebellum and the right mesial temporo-occipital region. ( **F** ) Follow-up imaging performed seven days later, showing a new vascular event in the mesial right occipital lobe (yellow arrow).

A 64-year-old male patient with hypertension and hyperlipidemia presented with sudden left eye vision loss. Imaging ( Figures 1 2 3 4 ) revealed recurrent posterior circulation infarcts and vessels occlusions. Angiography confirmed vertebral artery stump syndrome (VASS). An endovascular intervention successfully achieved complete recanalization without residual stenosis. The patient was discharged neurologically stable.

**Figure 2:**
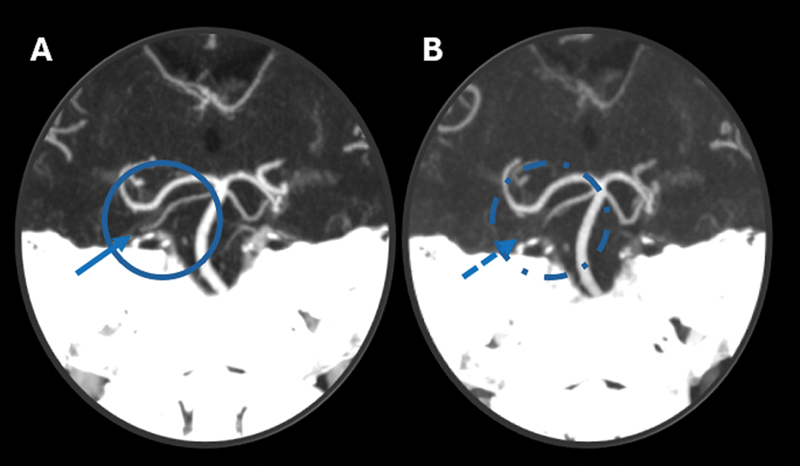
Computed tomography angiography (CTA) scan of the head, coronal maximum intensity projection (MIP) reconstruction with zoom-in of the vertebrobasilar system. ( **A,B** ) Images obtained seven days apart , showing an interval occlusion in the mid-to-distal segment of the right superior cerebellar artery (blue circles and arrows).

**Figure 3:**
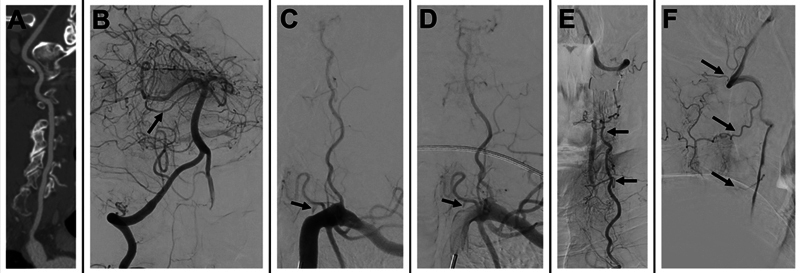
( **A** ) Computed tomography angiography (CTA) scan exhibiting normal right vertebral artery (VA). ( **B** ) Initial intracranial angiography of the right VA reveals normal intracranial flow and retrograde opacification of the intracranial left VA. Note that the right superior cerebellar artery is normal (arrow). ( **C,D** ) Left subclavian angiography depicts near-occlusion of the left VA origin with occlusion of its proximal V1 segment (arrows). Selective angiography of the left deep cervical artery in the frontal ( **E** ) and lateral ( **F** ) projections illustrate collateral supply to the distal V2 and V3 segments of the left VA via muscular anastomotic branches (arrows).

**Figure 4:**
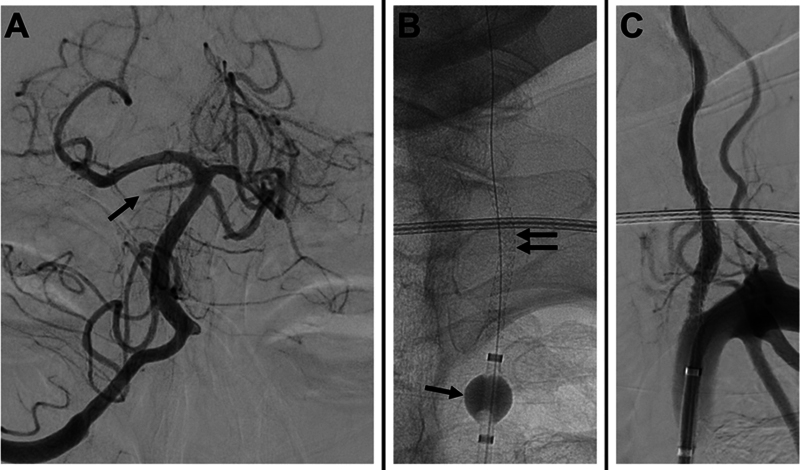
( **A** ) Repeat cerebral angiography 3 days later revealing a new occlusion of the right superior cerebellar artery (arrow). The decision was then made to attempt recanalization of the left VA. ( **B** ) A balloon guide catheter was positioned in the proximal left subclavian artery where the balloon was inflated to arrest antegrade flow and prevent distal embolization (single arrow); a balloon-expandable stent was then advanced and successfully deployed (double arrows). ( **C** ) The balloon of the balloon guide catheter was then deflated, and the final angiography confirms successful recanalization of the left VA.

Vertebral artery stump syndrome is a rare but treatable cause of recurrent posterior strokes, 1 2 3 with key mechanisms including stagnant blood flow leading to thrombus formation, propagation of embolic fragments from the occlusion's distal limit, and emboli introduced via collateral pathways. 4 This case highlights the importance of endovascular treatment in preventing neurological deterioration and improving outcomes.
